# Tubular cell-derived exosomal miR-150-5p contributes to renal fibrosis following unilateral ischemia-reperfusion injury by activating fibroblast *in vitro* and *in vivo*

**DOI:** 10.7150/ijbs.62478

**Published:** 2021-09-21

**Authors:** Xiangjun Zhou, Sheng Zhao, Wei Li, Yuan Ruan, Run Yuan, Jinzhuo Ning, Kun Jiang, Jinna Xie, Xiaobin Yao, Haoyong Li, Chenglong Li, Ting Rao, Weimin Yu, Fan Cheng

**Affiliations:** 1 Department of Urology, Renmin Hospital of Wuhan University, Wuhan, 430060, China.; 2 Department of Anesthesiology, Renmin Hospital of Wuhan University, Wuhan, 430060, China.

**Keywords:** renal fibrosis, unilateral ischemia-reperfusion, exosome, microRNA

## Abstract

Unilateral ischemia reperfusion injury (UIRI) with longer ischemia time is associated with an increased risk of acute renal injury and chronic kidney disease. Exosomes can transport lipid, protein, mRNA, and miRNA to corresponding target cells and mediate intercellular information exchange. In this study, we aimed to investigate whether exosome-derived miRNA mediates epithelial-mesenchymal cell communication relevant to renal fibrosis after UIRI. The secretion of exosomes increased remarkably in the kidney after UIRI and in rat renal tubular epithelium cells (NRK-52E) after hypoxia treatment. The inhibition of exosome secretion by Rab27a knockout or GW4869 treatment ameliorates renal fibrosis following UIRI *in vivo*. Purified exosomes from NRK-52E cells after hypoxia treatment could activate rat kidney fibroblasts (NRK-49F). The inhibition of exosome secretion in hypoxic NRK-52E cells through Rab27a knockdown or GW4869 treatment abolished NRK-49F cell activation. Interestingly, exosomal miRNA array analysis revealed that miR-150-5p expression was increased after hypoxia compared with the control group. The inhibition of exosomal miR-150-5p abolished the ability of hypoxic NRK-52E cells to promote NRK-49F cell activation *in vitro*, injections of miR-150-5p enriched exosomes from hypoxic NRK-52E cells aggravated renal fibrosis following UIRI, and renal fibrosis after UIRI was alleviated by miR-150-5p-deficient exosome *in vivo*. Furthermore, tubular cell-derived exosomal miR-150-5p could negatively regulate the expression of suppressor of cytokine signaling 1 to activate fibroblast. Thus, our results suggest that the blockade of exosomal miR-150-5p mediated tubular epithelial cell-fibroblast communication may provide a novel therapeutic target to prevents UIRI progression to renal fibrosis.

## Introduction

Unilateral renal ischemia-reperfusion injury (UIRI) often occurs in the perioperative period of nephron sparing partial nephrectomy, renal artery angioplasty, and renal transplantation, which could result in edema, hyperemia, fibrosis and other pathological changes 1. The historically safe duration of ischemia time was commonly thought to be 30 min 2. Longer ischemia time is associated with short- and long-term renal consequences, including acute renal injury and chronic kidney disease (CKD) 3. Renal fibrosis is a common pathological change in the progression of acute kidney injury to CKD. Therefore, one of the important ways to improve the long-term effect of UIRI on the kidney is to delay or reduce the process of fibrosis 4.

Renal tubular epithelial cells are the most serious site of IRI 5. The communication among injured tubular cells, interstitial fibroblasts, and inflammatory cells is mediated by exosome-elicited cellular responses 6, 7. Exosomes are extracellular vesicles with a diameter of 30-150 nm and composed of lipids, proteins, and nucleic acids 8. Exosome secretion, which involves the transportation of multivesicular bodies, is regulated by Rab guanosine triphosphatases, including Rab27a and Rab27b 9, 10. Exosome-mediated epithelial-mesenchymal cell communication is involved in the pathophysiological processes of many diseases, including renal injury and fibrosis 11, 12. Exosome-derived tubular epithelial cells could reverse IRI but aggravate renal fibrosis following IRI 13, 14, 15.

Exosomal miRNAs can be taken up by neighboring cells, subsequently modulate recipient cells, and play an important role in kidney disease progression 16. MiRNA is a small non-coding RNA with 18-25 nucleotides and is expressed in various tissues and organs of the body. MiRNA plays a negative regulatory role in post-transcriptional gene level expression; participates in cell activation, proliferation, transformation, apoptosis, and lipid metabolism; and is involved in the development of renal fibrosis 17. The miR-21, miR-192, and miR-200 families are highly expressed in renal fibrosis tissues and associated with the development of renal fibrosis 18.

However, whether exosomes mediate renal fibrosis following UIRI by shuttling miRNA has not yet been fully elucidated. In the present study, we showed how tubular cell-derived exosomal miRNAs are relevant to renal fibrosis. Our results stressed the importance of exosomal miRNA in renal fibrosis and provide a theoretical basis for understanding the mechanism of acute kidney injury progression to CKD.

## Materials and methods

### Animal model

C57 male mice (average weight 20 g) were purchased from HYcell Biotechnology (Wuhan, China), and Rab27a knockout mice (average weight 20 g) were purchased from Cyagen Biosciences (Guangzhou, China). A UIRI model was established according to a previous study 19. The mice were randomly allocated into the sham group and UIRI model group (n = 6 per group). The mice model of renal UIRI was established by clamping the pedicles for 30 or 60 min followed by reperfusion without contralateral nephrectomy. The kidneys of mice were removed at 2, 14, and 28 days after UIRI, and kidney tissues were collected for various analyses. Animal protocols were approved by Renmin Hospital of Wuhan University Animal Care and Use Committee. The methods were carried out in accordance with the approved guidelines.

### Cell culture and treatment

Rat renal tubular epithelial cells (NRK-52E) and fibroblasts (NRK-49F) were cultured with 10% fetal bovine serum and 1g/L glucose Dulbecco's modified Eagle medium (DMEM) at 37 °C with 5% CO_2_. NRK-52E cells were treated with 100 μmol/L CoCl_2_ for 24 h to induce hypoxia and incubated in exosome-free medium. The NRK-52E cells were pretreated with 10 μM GW4869. The NRK-49F cells were treated with 30 μg protein/mL exosomes isolated from hypoxia NRK-52E cells.

### Cell transfection

Anti-miR-150-5p LNA, miR-150-5p mimic, siRNA Rab27a, and their corresponding negative controls (NCs) were purchased from HYcell Biotechnology (Wuhan, China). NRK-52E cells with 60%-70% confluence was transfected using Lipofectamine 2000 according to the manufacturer's protocol.

### Exosome extraction

Fresh cell supernatant was transferred into a new sterile enzyme-free 15-mL centrifuge tube. Samples were centrifuged at 800 × g for 10min and 2000 × g for 20 min at 4 °C to remove cells and debris. The supernatant sample was transferred into a new 15-mL centrifuge tube and placed on ice for use. About half the volume of the exosome precipitation reagent was added and fully mixed in a vortex mixer. The samples were centrifuged at 10000 × g for 30 min, and the supernatant was removed carefully. The exosomes were resuspended in about 100 μL of PBS. Exosomes were isolated from kidney by mechanical sectioning and enzymatic digestion with collagenase and trypsin, followed by ultracentrifugation of the supernatant. The exosomes were stored at 2-8 °C for 1 week and in -80 °C.

### Nanoparticle tracking analysis (NTA)

Before sample detection, the sample chamber was cleaned with deionized water, and the ZetaView detection instrument (Particle Metrix, Meerbusch, Germany, PMX110) was calibrated with polystyrene microspheres (110 nm). The sample cell was cleaned with 1 × PBS buffer and diluted 200 times with 1 × PBS buffer for injection and detection. Size distributions and vesicle concentrations were assessed using the NTA software as previously described 20.

### Transmission electron microscopy (TEM)

Glutaraldehyde (1.25%, 11 μL) was added into 100 μL exosomes and then fixed overnight at 4 °C. The sample was dropped on the copper mesh of a carbon support membrane and placed for 3-5 min. Phosphotungstic acid (2%) was also dropped on the copper mesh of the carbon support membrane and placed for 2-3 min. Excess liquid was absorbed with filter paper and dried at room temperature. A transmission electron microscope was used to observe and collect images for analysis.

### miRNA microarray analysis

We used Affymetrix miRNA expression profiling chip to detect miRNA 21. Total RNA was extracted from the exosomes by appropriate methods and isolated using the mirVana™ miRNA Isolation Kit. Biotin was labeled with Genisphere FlashTag kit. The fluorescence scanning image of the chip was analyzed using the Affymetrix® GeneChip® Command Console® software.

### Fluorescence labeling of exosome

PKH26-labeled exosomes from the conditioned media of NRK-52E cells were incubated with NRK-49F for 24 h, exosome distribution was determined with the immunofluorescence *in vitro*. PKH26-labeled exosomes from hypoxic NRK-52E cells were injected into the mice via the tail vein, exosome distribution was determined with the animal Optical Imaging System *in vivo*.

### Western blot

Protein concentration was determined using a bicinchoninic acid kit. Electrophoresis was carried out at constant pressure with concentrated gel at 80 V and separation gel at 120 V until bromophenol blue reached the lower edge of the plate. The PVDF membrane was activated with methanol for 3 min before use. The time of membrane transfer in 300 Ma constant flow membrane was adjusted according to the molecular weight of the target protein. The first antibody SOCS1 (Boster Biological Technology co.ltd, PA1074), CD63 (Affinity Biosciences, AF5117), TSG-101 (Abcam, ab125011), fibronectin (Proteintech Group, Inc., 15613-1-AP), α-SMA (Boster Biological Technology co.ltd, BM0002) diluted with primary antibody diluent was added and incubated at 4 °C overnight. The diluted secondary antibody (ASPEN, AS1107) was added and incubated at room temperature for 30 min. Freshly mixed enhanced chemiluminescence solution (A: B = 1:1) was added to the protein side of the membrane and exposed in a dark room. The exposure conditions were adjusted according to different light intensities, developed, and fixed.

### Real-time polymerase chain reaction (PCR)

RNase-free water (100 μL) was added to dissolve the extracted total RNA. First cDNA was synthesized by EntiLink™ First-strand cDNA Synthesis Kit. Real-time fluorescent quantitative PCR was performed based on instructions of the StepOne™ Real-Time PCR Kit. Three complex holes were made in each sample by real-time PCR using EnTurbo™ SYBR Green PCR SuperMix Kit. The reaction procedure was as follows: pre-denaturation at 95 °C for 3 min and then 40 cycles each at 95 °C for 10 s, 58 °C for 30 s, and 72 °C for 30 s. The reaction system comprised 2 × Master Mix (5.0 μL), primer working solution (2.5 μm, 1.0 μL), template (1.0 μL), ddH_2_O (2.0 μL), and Rox (1.0 μL). The PCR primers are shown in Table 1.

### Luciferase reporter assay

The SOCS1 3'UTR carrying a miR-150-5p binding site was constructed by PCR and subsequently cloned into the pMIR-REPORT vector to construct the wild-type SOCS1 luciferase reporter. The wild-type SOCS1 construct and miR-150-5p mimic were co-transfected into HK-2 cells using Lipofectamine 2000. Luciferase activity was measured using the dual-luciferase assay.

### Histology, TUNEL, Sirius Red Staining, immunofluorescence staining and flow cytometry

According to routine protocols, the histological sections of the kidney (5 um) were dyed with hematoxylin-eosin (HE) to observe morphological changes. Apoptosis of renal tubular epithelial cells was examined by TUNEL staining and flow cytometry according to the manufacturer's instructions. Extracellular matrix accumulation in the kidney was detected by Masson trichrome staining, immunofluorescence staining and Sirius Red Staining according to routine protocols.

### Statistical analyses

All the results are expressed as mean ± standard deviation. SPSS 17.0 statistical software was used for statistical analysis. Comparisons between groups were made using one-way ANOVA, followed by Student-Newman-Kuels test. P<0.05 was considered significant difference.

## Results

### Increased renal injury and fibrosis are associated with prolonged ischemic time after UIRI

**Figure 1:**
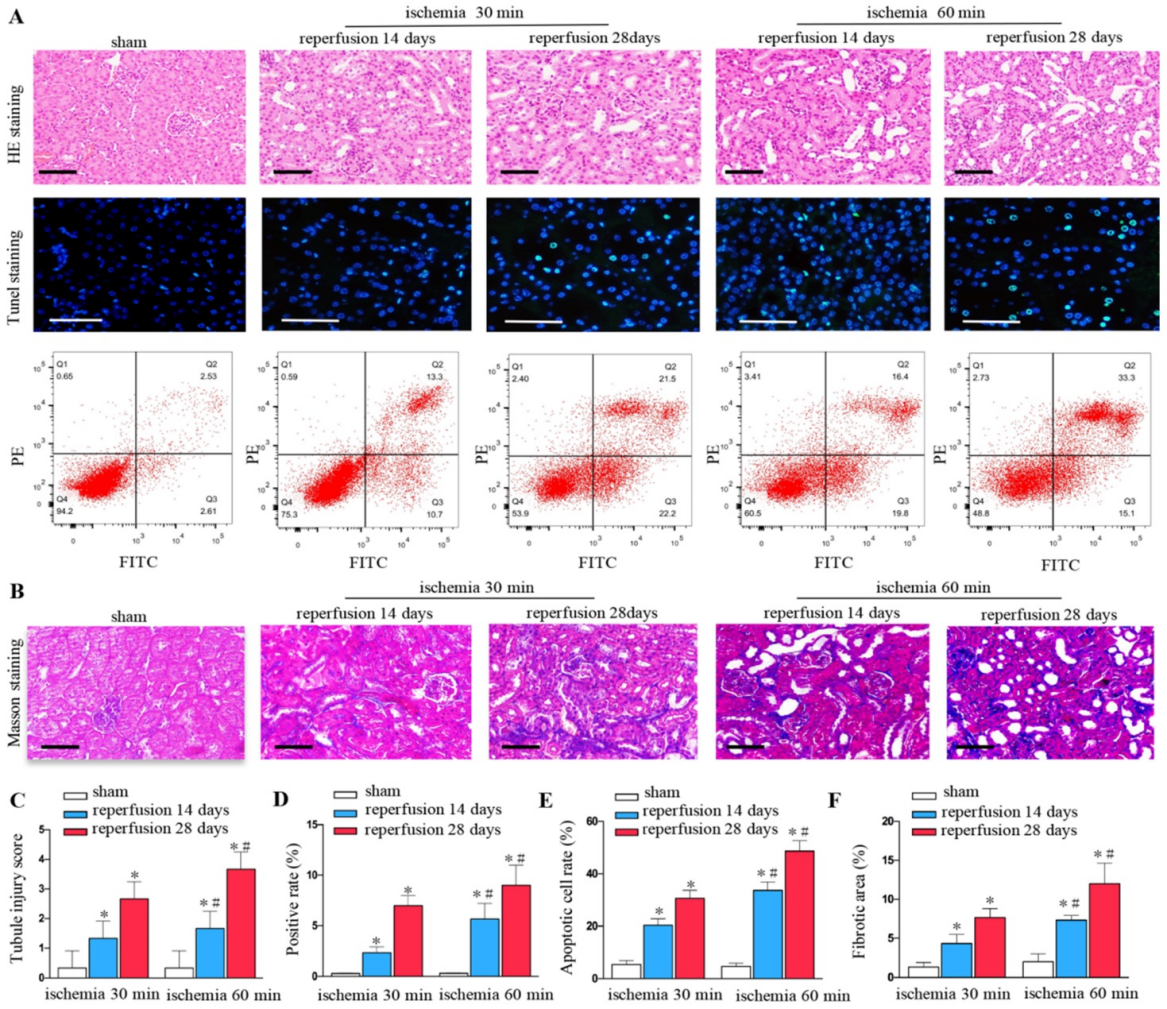
** Increased renal injury and fibrosis are associated with prolonged ischemic time in UIRI. (A)** HE, TUNEL staining and flow cytometry of kidney tissues from the sham and UIRI groups at 14 days, 28 days (n=6). **(B)** Area of collagen in different groups as indicated by Masson staining. **(C)** Evaluation of tubular injury scores by HE staining in the sham and UIRI groups at 14 days, 28 days. **(D)** Evaluation of positive cell apoptotic rate by TUNEL staining. **(E)** Evaluation of apoptotic rate by flow cytometry. **(F)** Quantitative determination of collagen area in different groups. ^*^P < 0.05 versus sham controls; ^#^P < 0.05 versus ischemia 30 minutes.

Renal ischemia-reperfusion injury can lead to the destruction of renal tissue structure, renal tubule expansion, cell exfoliation and necrosis, and gradually evolve into fibrosis. We found that unilateral ischemia for 60 min and reperfusion is remarkably more serious than ischemia for 30 min and reperfusion at days 14 and 28 after UIRI as shown by HE staining (Figures 1A and C). The rate of cell apoptosis significantly increased at ischemia for 60 minutes, reperfusion for 14 days, 28 days by TUNEL staining (Figures 1A and D) and flow cytometry (Figures 1A and E). Ischemia for 60 min produced more extracellular matrixes than ischemia for 30 min at days 14 and 28 as determined by Masson staining (Figures 1B and F).

### Renal exosome secretion increases following UIRI

**Figure 2:**
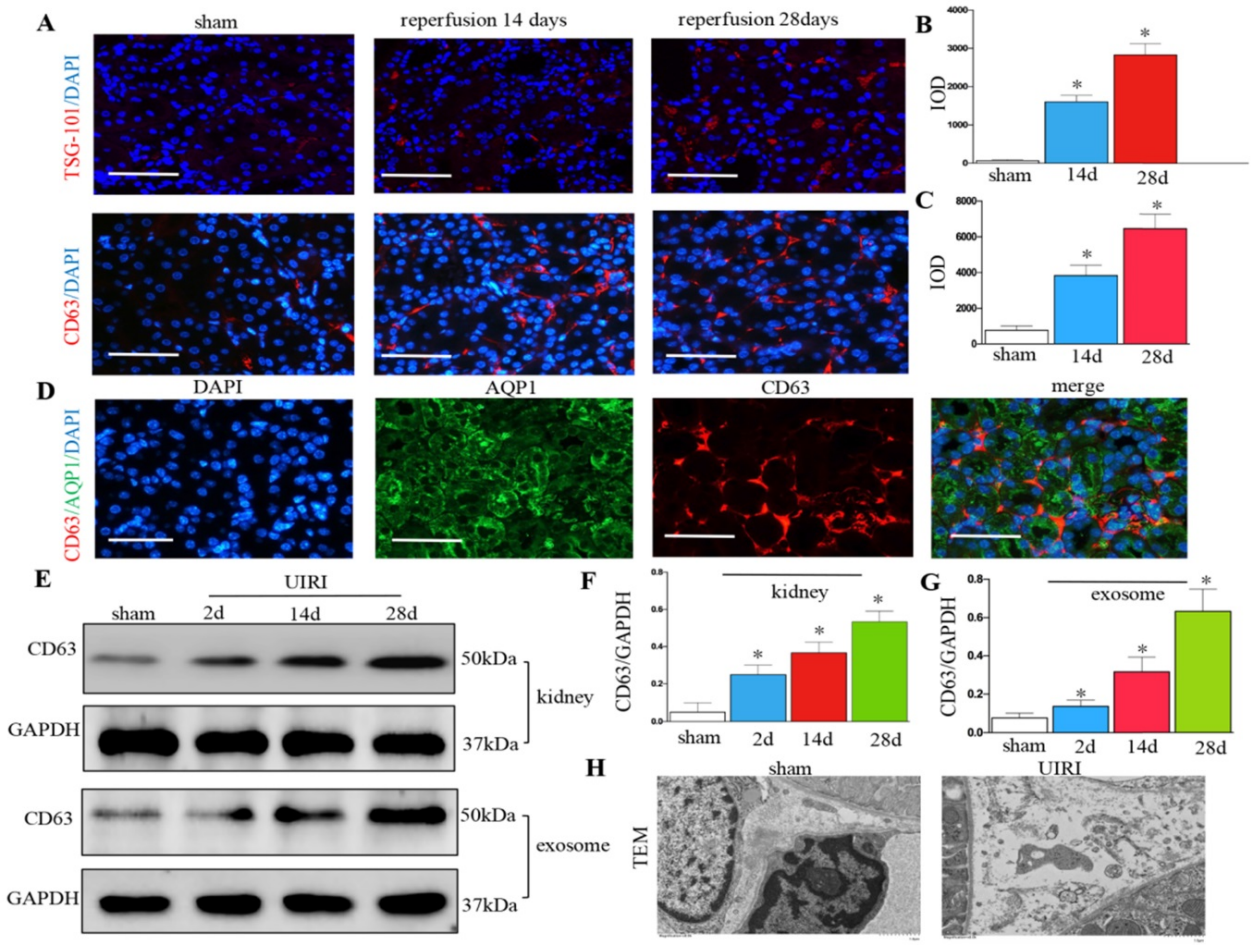
** Increased secretion of exosomes in the kidney following UIRI. (A)** Immunofluorescence staining for CD63 and TSG-101 (red). **(B)** Integrated optical density (IOD) of TSG-101. **(C)** Integrated optical density (IOD) of CD63. **(D)** Double immunofluorescence staining for CD63 (red) and AQP-1 (green), respectively. **(E)** Western blotting of CD63 in kidney tissue and exosome at different time points after UIRI (n=6). **(F)** Quantitative of CD63 in kidney. **(G)** Quantitative of CD63 in exosome. **(H)** TEM image of the exosomes in the kidney tissue after UIRI. ^*^P < 0.05 versus sham.

We detected the production of exosomes in UIRI. Immunofluorescence staining of CD63 and TSG-101 (exosomal marker) showed exosome secretion increased in the kidney after UIRI (Figures 2A-C). Immunostaining for CD63 and AQP-1 (tubular cell marker) revealed that exosomes were predominantly localized around the renal tubular epithelium (Figure 2D). CD63 from kidney tissue continued to increase from 2 days to 28 days after UIRI (Figures 2E and F). CD63 from the isolated renal exosomes also continued to increase from 2 days to 28 days after UIRI (Figures 2E and G). Moreover, many extracellular vesicles had an exosome diameter of 30-150 nm after UIRI as shown by TEM (Figure 2H).

### Inhibition of exosome secretion by Rab27a knockout and inhibitor GW4869 treatment prevents renal fibrosis after UIRI *in vivo*

**Figure 3:**
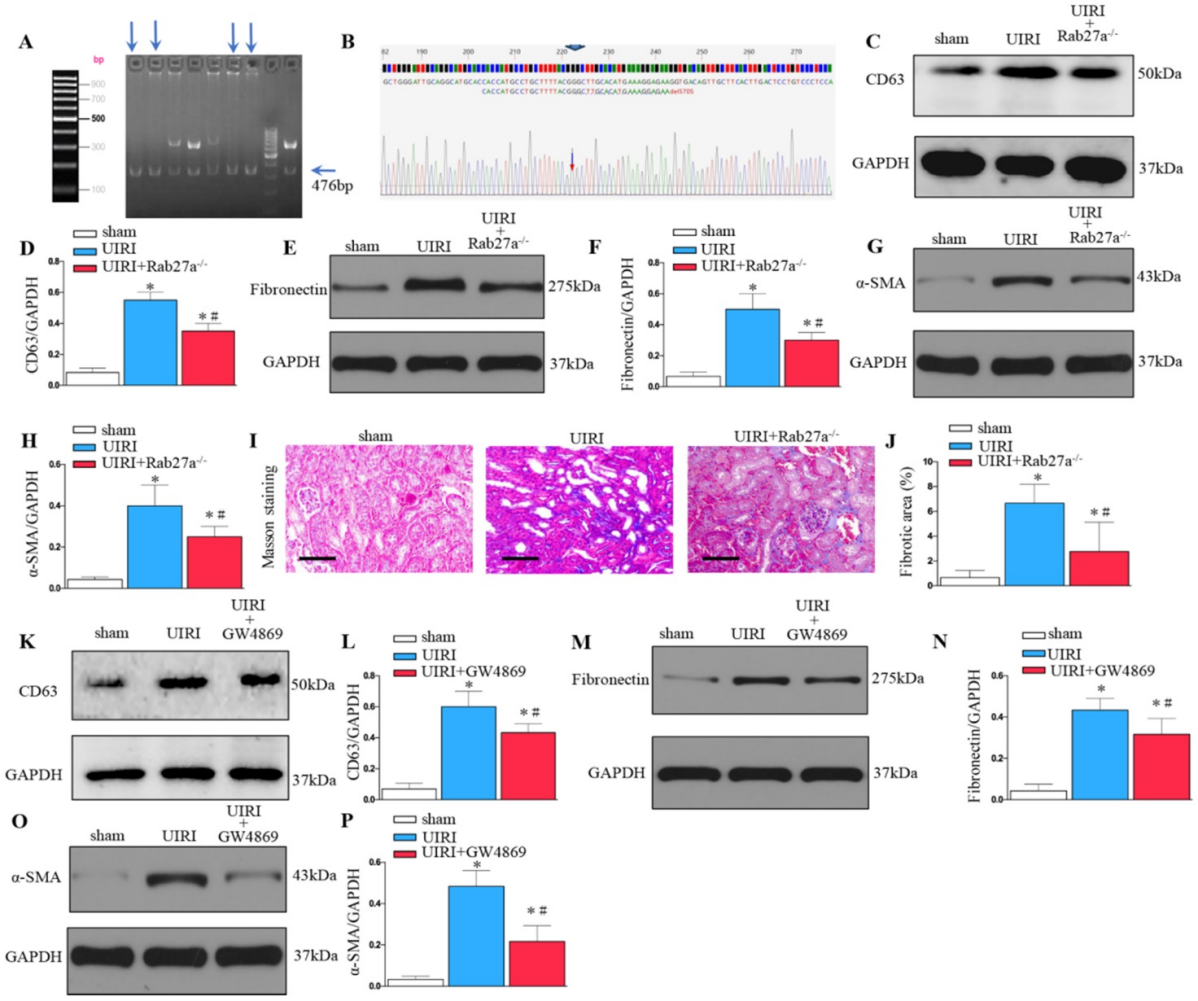
** Inhibition of exosome secretion by Rab27a knockout and inhibitor GW4869 prevents renal fibrosis *in vivo*. (A, B)** Rab27a knockout as confirmed by PCR screening (Targeted allele: 476 bp) and sequencing confirmation. **(C)** Western blot analysis of CD63 in kidney after Rab27a knockout. **(D)** Quantitative data of CD63 are presented. **(E)** Western blotting of fibronectin in kidney. **(F)** Quantitative data of fibronectin are presented. **(G)** Western blotting of α-SMA in kidney. **(H)** Quantitative data of α-SMA are presented. **(I)** Area of collagen in different groups as indicated by Masson staining. **(J)** Quantitative of kidney fibrotic area in different groups. **(K)** Western blotting of CD63 in kidney after GW4869 treatment (n=6). **(L)** Quantitative data of CD63 are presented. **(M)** Western blotting of fibronectin in kidney. **(N)** Quantitative data of fibronectin are presented. **(O)** Western blotting of α-SMA in kidney. (P) Quantitative data of α-SMA are presented. ^*^P < 0.05 versus sham; ^#^P < 0.05 versus UIRI.

As shown in Figures 3A and B, Rab27a knockout mice (Rab27a^-/-^) were successfully constructed and confirmed. Rab27a knockout reduced exosome secretion after UIRI (Figure 3C and D). Western blotting confirmed that knockout of Rab27a ameliorated renal fibrosis after UIRI by inhibiting fibroblast activation (α-SMA) and fibronectin deposition (Figures 3E-H). Similarly, Masson staining confirmed that knockout of Rab27a decreased the deposition of collagen after UIRI (Figures 3I and J).

To further study the role of exosomes in renal fibrosis, we inhibited the release of exosomes by inhibitor GW4869 treatment. As shown in Figure 3K and L, GW4869 treatment reduced exosome secretion after UIRI. Western blotting confirmed that GW4869 treatment inhibited extracellular matrix deposition and fibroblast activation after UIRI (Figures 3M-P).

### Increase in tubular cell-derived exosomes after hypoxia treatment promotes fibroblast activation

**Figure 4:**
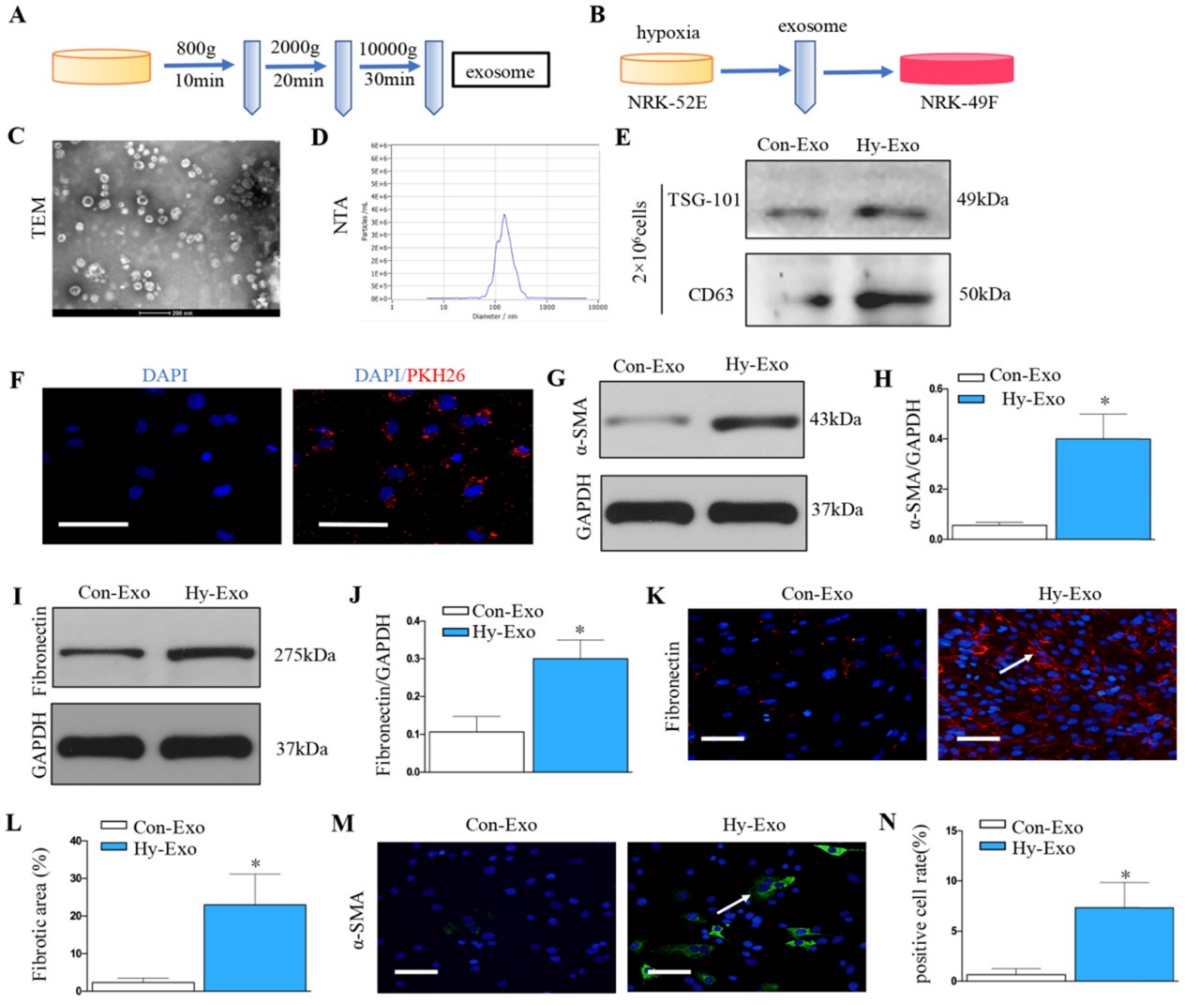
** Increased tubular cell-derived exosomes after hypoxia treatment mediate fibroblast activation. (A)** Diagram shows the exosomes were isolated from conditioned media by ultra-centrifugation. **(B)** Experimental design shows exosomes were extracted from NRK-52E cells treated without (Con-Exo) or with hypoxia (Hy-Exo) incubated with NRK-49F cells (n=3). **(C)** TEM image of the exosomes isolated from hypoxic NRK-52E cells. **(D)** NTA of exosomes from hypoxic NRK-52E cells. **(E)** Western blotting of CD63 and TSG-101 as exosome marker. **(F)** Fluorescent staining image of NRK-52E cell-derived exosome being taken up by NRK-49F cells. **(G)** Western blotting of α-SMA after Hy-Exo treatment. **(H)** Quantitative of α-SMA are presented. (I) Western blotting of fibronectin after Hy-Exo treatment. **(J)** Quantitative of fibronectin are presented. **(K)** The slides of cell were subjected to immunostaining for fibronectin. Arrows indicate positive staining. **(L)** Representative micrographs show fibronectin expression in different groups as indicated. **(M)** The slides of cell were subjected to immunostaining for α-SMA. Arrows indicate positive staining. **(N)** Representative micrographs show α-SMA expression in different groups as indicated. ^*^P < 0.05 versus Con-Exo.

Exosomes was extracted from NRK-52E cells treated without (Con-Exo) or with hypoxia (Hy-Exo) by ultra-centrifugation (Figure 4A) and then incubated with NRK-49F cells (Figure 4B). The TEM and NTA results display the shapes and sizes of the isolated exosomes (Figures 4C and D). Hypoxia treatment increased exosomes secretion with the same number of NRK-52E cells as demonstrated by Western blot of CD63 and TSG-101 (maker of exosome) (Figure 4E). We labeled the exosomes from the condition medium of NRK-52E cells with PKH26, a lipophilic fluorescent dye for tracing. PKH-26 labeled exosomes incubated with NRK-49F cells were up-taken by fibroblasts (Figure 4F). Furthermore, western blotting confirmed that exosomes isolated from hypoxia-treated NRK-52E cells could induce extracellular matrix deposition and fibroblast activation (Figures 4G-J). Similarly, immunostaining confirmed that exosomes isolated from hypoxia-treated NRK-52E cells could induce fibronectin deposition and fibroblast activation (Figures 4K-N).

### Inhibition of tubular cell-derived exosome by Rab27a knockdown and GW4869 treatment attenuates fibroblast activation *in vitro*

**Figure 5:**
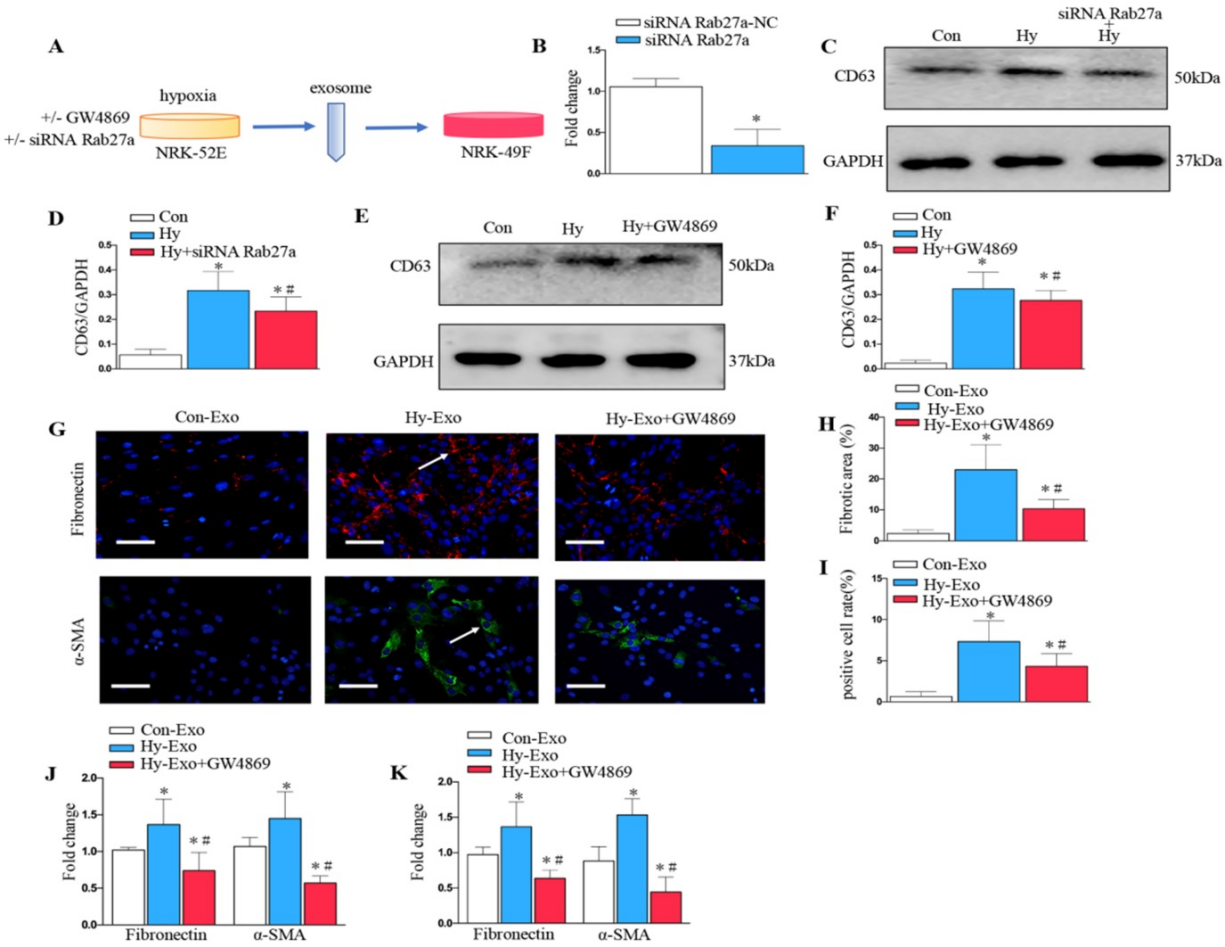
** Inhibition of exosome secretion by Rab27a knockdown and inhibitor GW4869 attenuates fibroblast activation *in vitro*. (A)** Experimental design shows exosome from hypoxia NRK-52E cells with GW4869 or siRNA Rab27a treatment to stimulate NRK-49F cells. **(B)** PCR detection of Rab27a after transfection with siRNA Rab27a. **(C)** Western blotting of CD63 in exosome after Rab27a knockdown. **(D)** Quantitative of CD63 with siRNA Rab27a treatment are presented. **(E)** Western blotting of CD63 in exosome after GW4869 treatment. **(F)** Quantitative of CD63 with GW4869 treatment are presented. n=3, ^*^P < 0.05 versus Con; ^#^P < 0.05 versus Hy. **(G)** The slides of cell were subjected to immunostaining for α-SMA and fibronectin. Arrows indicate positive staining. **(H)** Representative micrographs show fibronectin expression in different groups as indicated. **(I)** Representative micrographs show α-SMA expression in different groups as indicated. **(J)** Fold changes in the expression levels of α-SMA and fibronectin with siRNA Rab27a treatment. **(K)** Fold changes in the expression levels of α-SMA and fibronectin with GW4869 treatment. n=3, ^*^P < 0.05 versus Con-Exo; ^#^P < 0.05 versus Hy-Exo.

We further analyzed the role of exosomes in epithelial cell-fibroblast cell communication. Exosomes from the NRK-52E cells subjected to hypoxia or treated with GW4869 or siRNA Rab27a were collected to stimulate NRK-49F (Figure 5A). As shown in Figure 5B, Rab27a was knocked down after transfection with siRNA Rab27a. Knockdown of Rab27a and GW4869 treatment inhibited secretion of exosome as demonstrated by Western blot of CD63 (Figure 5C-F). The blockade of exosome secretion from tubular cells subjected to hypoxia by GW4869 treatment inhibited fibroblast activation and fibronectin deposition as demonstrated by fibronectin, α-SMA (Figures 5G-I). Similarly, PCR confirmed that the inhibition of exosome secretion from hypoxic tubular cells by Rab27a siRNA or GW4869 treatment inhibited fibroblast activation and extracellular matrix accumulation as manifested by α-SMA and fibronectin (Figures 5J and K).

### Tubular cell-derived exosomal miR-150-5p mediates fibroblast activation by targeting suppressor of cytokine signaling 1 (SOCS1) *in vitro*

**Figure 6:**
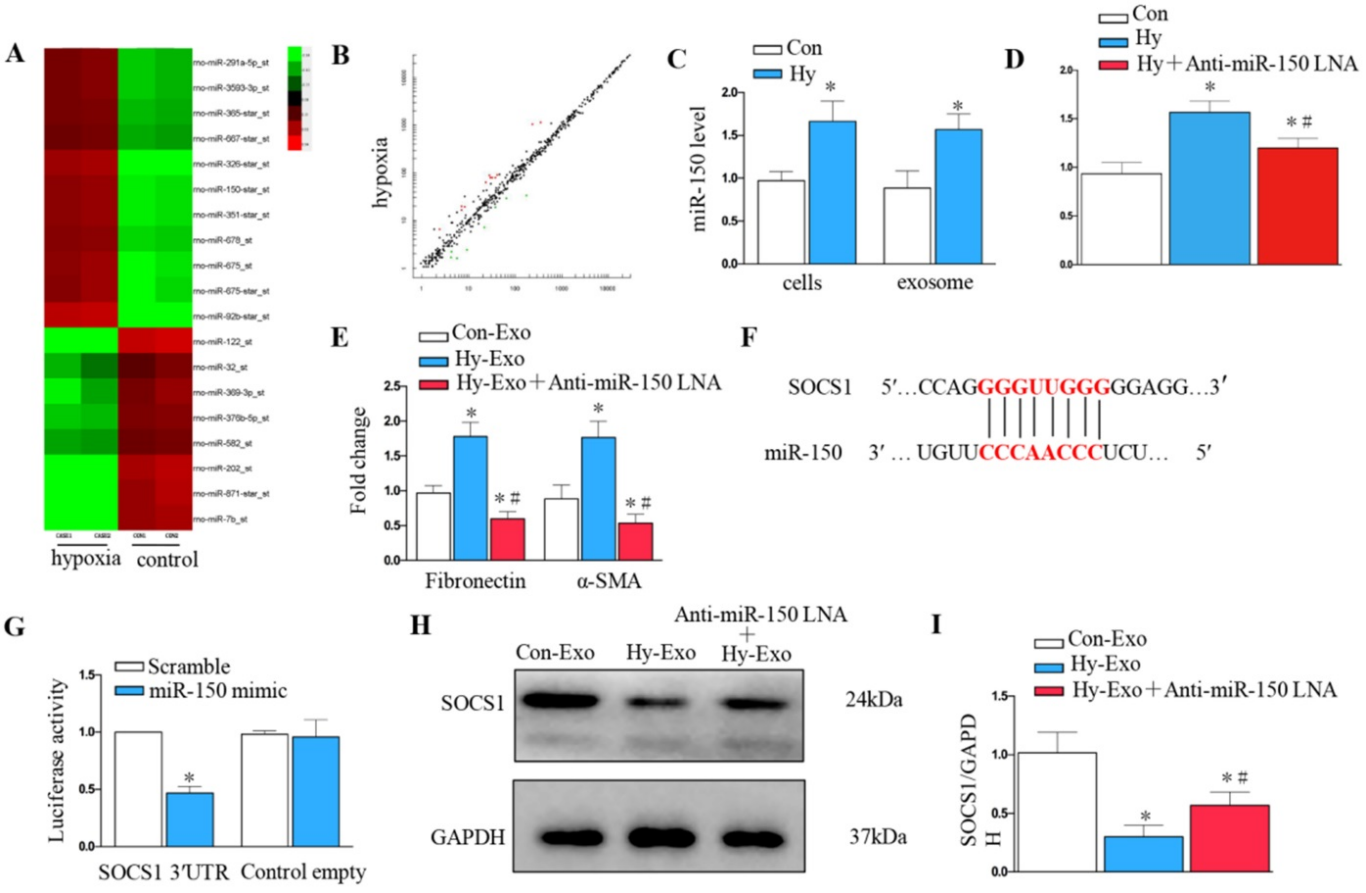
** Tubule cell-derived exosomal miR-150-5p mediate fibroblast activation by SOCS1 *in vitro*. (A)** Exosomal miR assay analysis of NRK-52E cells after hypoxia treatment. **(B)** Scatter plot showed exosomal miR changes following hypoxia treatment. **(C)** PCR detection of miR-150-5p in exosomes and NRK-52E cells after hypoxia treatment. **(D)** PCR detection of miR-150-5p in NRK-52E cells after Anti-miR-150 LNA treatment. **(E)** PCR detection of α-SMA and fibronectin. **(F)** Potential miR-150 binding site in 3'-UTR of SOCS1 mRNA. **(G)** Luciferase activity in NRK-49F cells transfected with negative control or miR-150-5p mimic together with reporter vector containing SOCS1-mut binding sequences. **(H)** Immunoblot analysis of SOCS1 expression. **(I)** Densitometry analysis of SOCS1 expression. n=3, ^*^P < 0.05 versus Con-Exo; ^#^P < 0.05 versus Hy-Exo.

We further explored whether tubular cell-derived exosomal miRNA mediates fibroblast proliferation and activation. Exosomal miR assay analysis was performed on NRK-52E cells after hypoxia treatment (Figure 6A), about 20 microRNAs showed consistent changes in expression following hypoxia treatment: 12 were increased, whereas 8 decreased (Figure 6B). We further confirmed miR-150-5p was induced in renal tubular epithelial cells and exosomes after hypoxia treatment by PCR (Figure 6C). We knocked down miR-150-5p in NRK-52E cells by transfecting anti-miR-150-5p-LNA to determine the role of exosomal miR-150-5p in fibroblast activation (Figure 6D). As shown in Figure 6E, MiR-150-5p knockdown in the exosomes of NRK-52E cells prevented fibronectin and α-SMA expression in fibroblasts.

We further investigated the possible mechanism of how tubular cell-derived exosomal miR-150-5p mediates fibroblast activation. Targetscan Software predicted that the 3′-untranslated region (UTR) of the *SOCS1* gene was the conserved binding site of miR-150 (Figure 6F). To verify the relationship between miR-150-5p and SOCS1, a wild-type SOCS1 (SOCS1-WT) luciferase reporter construct carrying the SOCS1 3′UTR was constructed. After the transfection of a miR-150-5p mimic with SOCS1-WT into NRK-49F cells, miR-150-5p mimics suppressed the luciferase activity in SOCS1-miR-150-5p-transfected cells compared to the control group (Figure 6G). Consistently, hypoxia epithelial cells secreted exosomes downregulated SOCS1 in fibroblasts, while inhibiting the expression of miR-150-5p in exosomes upregulated SOCS1expression in fibroblasts (Figures 6H and I).

### Tubular cell-derived exosomal miR-150-5p promote renal fibrosis by inhibiting SOCS1 after UIRI *in vivo*

**Figure 7:**
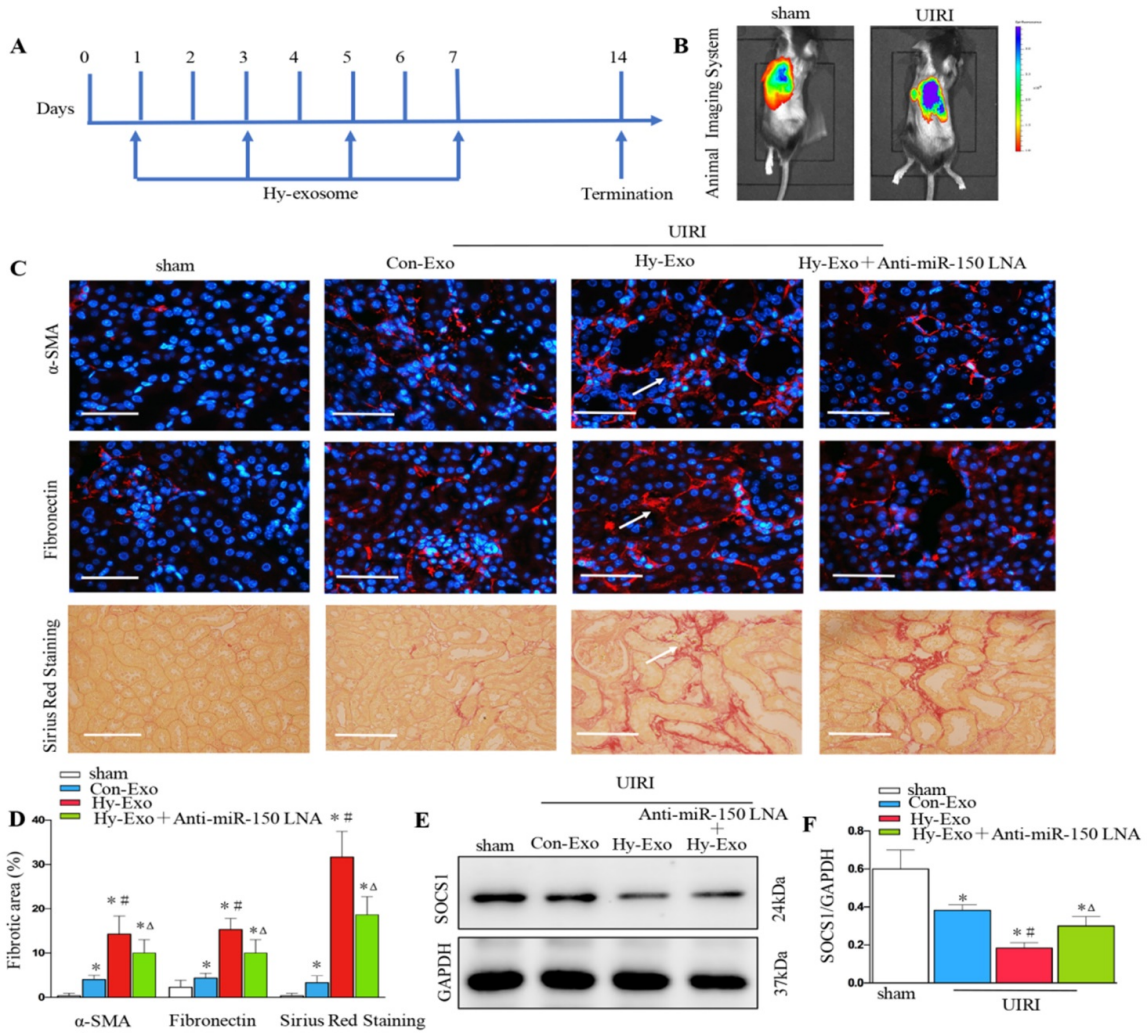
** Tubular cell-derived exosomes promote renal fibrosis by inhibiting SOCS1 after UIRI *in vivo*. (A)** Diagram shows NRK-52E derived exosomes were injected i.v. at days 1,3,5 and 7 after UIRI. **(B)** PKH26-labeled exosomes in kidney with UIRI kidney as shown by an *in vivo* imaging system. **(C)** Paraffin sections were subjected to fibronectin and α-SMA immunostaining, Sirius Red Staining (n=6). Arrows indicate positive staining. **(D)** Representative micrographs showed collagen deposited for α-SMA and fibronectin immunostaining and Sirius Red Staining in different groups as indicated. **(E)** Expression of SOCS1 were examined by Western blot analysis after exosome injection. **(F)** Densitometry analysis of SOCS1 expression. ^*^P < 0.05 versus sham; ^#^P < 0.05 versus Con-Exo; ^Δ^P < 0.05 versus Hy-Exo.

We injected exosomes isolated from hypoxia-treated NRK-52E cells into the kidney by tail vein to investigate their role in renal fibrosis after UIRI. UIRI mice were given the same amounts of exosome at 1, 3, 5 and 7 days (Figure 7A). We first found PKH26 labeled tubular cell exosomes in the kidney by *in vivo* imaging technology (Figure 7B). Interestingly, the exosomes isolated from hypoxia-treated cells increased the extracellular matrix at 14 days after UIRI as determined by immunostaining staining of fibronectin and α-SMA and Sirius Red Staining, while inhibiting the expression of miR-150-5p in tubule cell-derived exosome inhibited fibroblast activation and extracellular matrix production (Figures 7C and D). We further found that exosomes secreted by hypoxic epithelial cells downregulated SOCS1 in the kidney following UIRI and the inhibition of miR-150-5p expression in exosomes also upregulated SOCS1 in the kidney following UIRI (Figures 7E and F).

## Discussion

Our study found that the exosome secretion of renal tubular epithelial cells under UIRI or hypoxia was remarkably increased. The exosomes secreted by renal tubular epithelial cells under hypoxia could activate fibroblasts and aggravate renal fibrosis. On the contrary, the inhibition of the exosome secretion of these renal tubular epithelial cells remarkably inhibited fibroblast activation and delayed renal fibrosis. The exosomes rich in miR-150-5p secreted by hypoxic renal tubular epithelial cells are one of the main reasons for the activation of fibroblasts and the aggravation of renal fibrosis. Thus, tubular cell-derived exosomal miR-150-5p may play a biological role in fibroblasts activation.

Exosomes are secreted by a variety of living cells; carry a variety of important information, such as proteins, lipids, and RNA; and play an important role in the material and information transmission between cells 22. Exosome secretion is triggered by extracellular or intracellular stress 23, 24. Hypoxia is a severe cellular stress that can regulate the release of exosomes 25. Exosomes have been investigated in different types of hypoxic diseases and found to have many effects from pathology to protection, including myocardial infarction, renal ischemia-reperfusion-induced acute kidney injury, and hypoxic tumors 26. Cancer cells produce more exosomes under hypoxic conditions than parental cells under normoxic conditions 27. Pulmonary artery endothelial cells 28, adipocytes 29, and cardiac progenitor cells 30 also release more exosomes under hypoxic conditions. In our previous study, hypoxia (1% O_2_) did not change the average sizes of exosomes secreted by tubular epithelial cells but considerably increased exosome production in a time-dependent manner 31. In the present study, exosome secretion increased after renal UIRI and CoCl_2_-induced hypoxia. The results of this study are consistent with previous results, that is, prolonged ischemia-reperfusion results in more exosome secretion.

Exosomes have diverse functions, which depend on the source. Exosomes mediate cell-cell communication related to renal fibrosis 32. The exosomes secreted by damaged kidney cells can be transported to other normal kidney cells and activate fibroblasts, which promotes renal fibrosis and leads to a vicious cycle of renal injury 33. Tubular cell-derived exosomes from unilateral ureteral obstruction and IRI models of kidney disease play an important role in fibroblast activation; damaged tubular epithelial cells produce and release exosomes to promote the proliferation of adjacent fibroblasts, which manifests in the production of α-SMA and collagen I 34. Our study also confirmed that the exosomes secreted by renal tubular epithelial cells under UIRI or hypoxia could activate fibroblasts and aggravate renal fibrosis. On the contrary, the inhibition of the exosome secretion of renal tubular epithelial cells UIRI or hypoxia remarkably inhibited the activation of fibroblasts and delayed the process of renal fibrosis.

Exosomes contain mRNA and miRNA, which can be delivered to another cell and become functional in renal injury and fibrosis. Exosomal miR-23a derived from hypoxic renal tubular epithelial cells activates macrophages to promote tubulointerstitial inflammation 35. Exosomal miR-20a-5p derived from hypoxic renal tubular epithelial cells protects against acute tubular injury 36. MiRNAs, including miR-192, miR-320, and miR-34, are involved in the mechanism of how exosomes mediate renal fibrosis 37, 38, 39. In the present study, we finally selected exosomal miR-150-5p as the target of our further study. First, miRNA profiling of exosomes extracted from renal tissue samples with or without ischemia-reperfusion injury revealed that miR-150 was markedly increased as a profibrotic molecule 40. Second, our pre-experiments detected by PCR and miRNA profiling of exosomes from epithelial cells with hypoxia injury indicated that miR-150-5p was one of the most obvious change among miRNAs. Importantly, miR-150 is a potential novel therapeutic agent for tubulointerstitial fibrosis. LNA-anti-miR-150 ameliorates renal fibrosis initiated by glomerular injury in mouse models of lupus nephritis and focal segmental glomerulosclerosis 41. Our study also confirmed that the exosomal miR-150-5p secreted by renal tubular epithelial cells under hypoxia could activate fibroblasts and aggravate renal fibrosis following UIRI.

In the present study, we have identified SOCS1 as a direct target of miR-150-5p. SOCS1 is a member of the suppressor of cytokine signaling (SOCS) family of proteins, which negatively regulates JAK/STAT pathway activation and affects multiple cellular activation 42. Previous animal studies from our group demonstrated that the therapeutic targeting of the SOCS family prevents kidney tissue injury 43. The downregulation of SOCS1 is correlated with the amplified production of pro-inflammatory factors and cell apoptosis, which participate in renal fibrosis in lupus nephritis 44. SOCS1 is downregulated in epithelial cells and fibroblasts involved in renal fibrosis 45. Our study confirmed tubule cell-derived exosomal miR-150-5p promote fibroblast activation by SOCS1 after UIRI.

**Figure 8:**
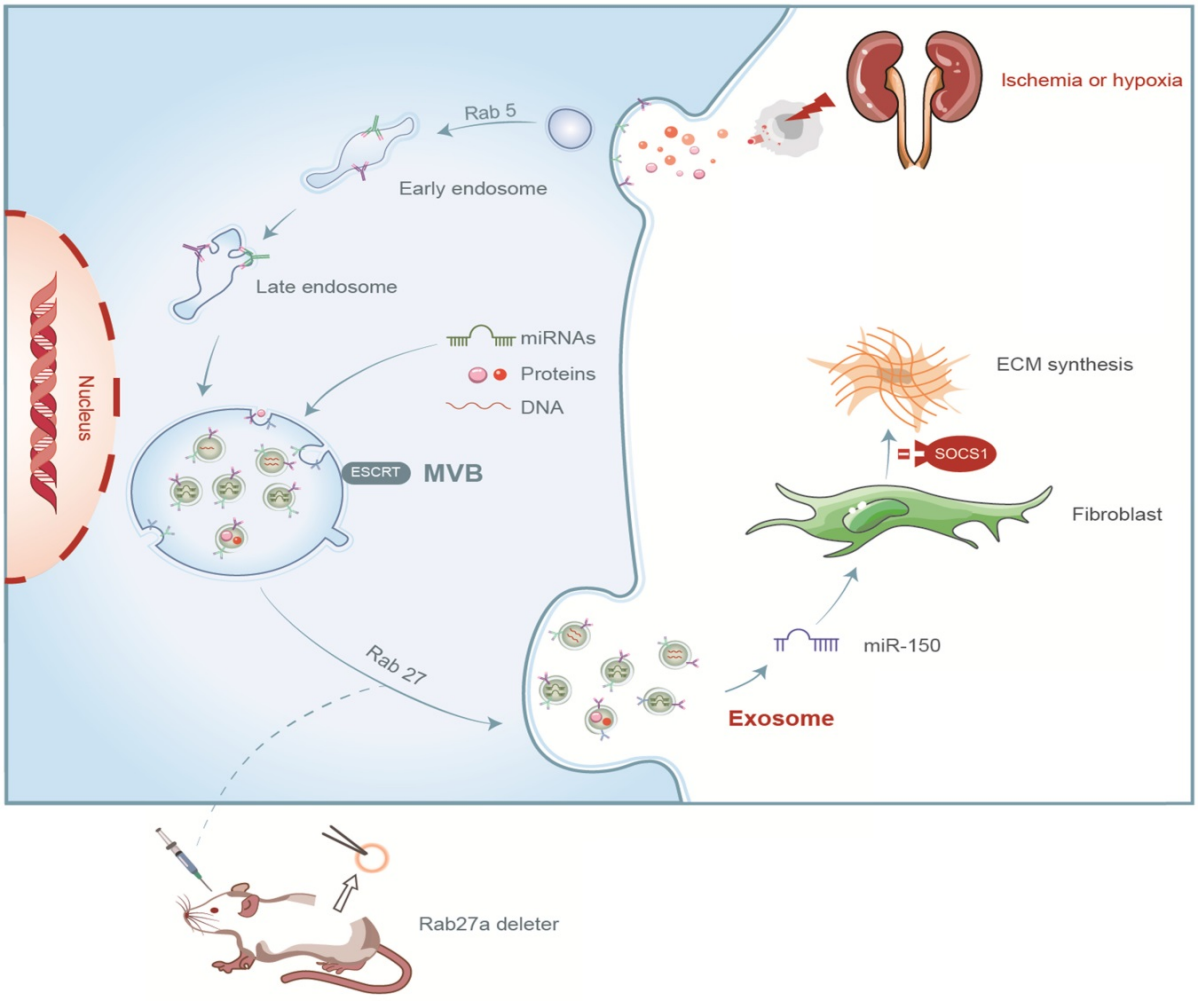
Summary of the role of exosome in renal fibrosis after UIRI.

In summary, we demonstrated that tubular cell-derived exosomes play an important role in renal fibrosis (Figure 8). We found that the production of exosomes that contain miR-150-5p increased after UIRI or hypoxia. The delivery of tubular cell-derived exosomal miR-150-5p promoted fibroblast activation by SOCS1. These results introduce a new mechanism of how cell-cell communication is mediated by exosomes in UIRI and that exosomes could be a new area that could retard or slow down the progression of renal fibrosis.

## Figures and Tables

**Table 1:** PCR primer sequence

Primer name	Base sequence (5'- 3')
**R-GAPDH**	
sense	GCCAAGGTCATCCATGACAAC
antisense	GTGGATGCAGGGATGATGTTC
**R-FN**	
sense	GCCCTTACAGTTCCAAGTTCC
antisense	AAACCGTGTAAGGGTCAAAGC
**R-α-SMA**	
sense	AGCATCCGACCTTGCTAACG
antisense	CCAGAGTCCAGCACAATACCAG
**R-Rab27a**	
sense	GCAGAGAAGTATGGAATCCCCT
antisense	GAAGTATGGCCATTGGACCG
**M-Rab27a**	
sense	CATCTCCCTGGTCTCTATAAAATC
antisense	ACATCCATAAAACATATTCCCCTC
